# Fertile Ground in the OR: Rewriting the Narrative on Family in Plastic Surgery Training

**DOI:** 10.7759/cureus.116367

**Published:** 2026-09-16

**Authors:** Nicole K Le, Meredith G Moore, Michael Harrington

**Affiliations:** 1 Plastic and Reconstructive Surgery, Stanford University, Palo Alto, USA; 2 Plastic Surgery, University of South Florida, Tampa, USA

**Keywords:** family-friendly training, parenthood, plastic surgery residency, residency, surgical education

## Abstract

This editorial discusses the possibility of family life and plastic surgery residency successfully coexisting when training programs intentionally foster supportive cultures, flexible policies, and equitable parental leave. Drawing on personal experiences from a highly family-friendly program, together with emerging literature on pregnancy, parenthood, childcare, and career advancement in surgery, we challenge the notion that residents must choose between surgical excellence and family life. Some practical strategies, including shared workload, flexible scheduling, lactation support, peer mentorship, childcare assistance, and visible role models, may help address the barriers that resident parents face while supporting professional development and personal well-being. Ultimately, we contend that programs supporting residents’ family goals strengthen the specialty by promoting wellness, inclusivity, and long-term retention, particularly of women in academic plastic surgery.

## Editorial

Our program director recently opened an email greeting us from “the most fertile plastic surgery program in the universe,” followed by photos of two residents’ newborns-delivered on the same day, in the same hospital. One former resident even welcomed triplets, and collectively, the authors of this letter have six children. Although few would call parenting during surgical training “easy,” our experience demonstrates that family life and residency can coexist productively and even synergistically when programs foster the right environment.

Plastic surgery training coincides with a period of reproductive life in which decisions about childbearing may carry important personal and medical considerations. Delaying family building until after residency may bring its own difficulties, including infertility, postponed personal goals, and older potential grandparents less able to assist. Surveys of US female surgeons have reported higher rates of infertility and pregnancy complications compared with female nonsurgeon partners, and greater operative exposure during the third trimester has been associated with increased risk of major pregnancy complications. In one national survey, female surgeons reported infertility more frequently than nonsurgeon partners, while those operating 12 or more hours per week during the third trimester had higher odds of major pregnancy complications. These findings do not imply that pregnancy during surgical training is inherently unsafe; rather, they underscore the importance of individualized counseling, appropriate occupational accommodations, and institutional support for residents who choose to become parents during training [1,2].

Our program director is a strong supporter of family planning whenever it is right for the resident and their significant other. This stems from his and his wife’s own infertility issues due to “waiting for the right time.” As he learned the hard way, there is never a “right time” during surgical training, as each advancing year holds its own challenges. Even after graduation, launching a viable practice and adhering to the standards of the board collection period can take its own toll. He always says that as an educator, he teaches the residents to learn from his mistakes, including those outside of surgical education.

Drawing from our experience at our institution, we offer several practical observations.

Work-life balance

Success requires teamwork, efficiency, and flexibility. Responsibilities such as rounds, surgery, clinic, and documentation are best shared transparently, with acknowledgment that not all rotations are equally demanding. Attendance at medical and pediatric appointments should be supported when feasible. This need is supported by survey data demonstrating that childcare remains a substantial financial and logistical burden for plastic surgery residents. Wallace et al. found that all respondents with children reported childcare as a financial burden, while 63% reported that their programs lacked sufficient flexibility to accommodate childcare needs [2,3]. More broadly, a national needs assessment of residents and fellows found that childcare access, affordability, parental leave, and lactation support were among the most frequently identified challenges for trainee parents [4].

Supportive policies

Parental leave policies should meet Accreditation Council for Graduate Medical Education (ACGME) and American Board of Plastic Surgery (ABPS) requirements while remaining humane and equitable [5]. Current ACGME institutional requirements provide for a minimum of six weeks of approved medical, parental, and caregiver leave during training, with 100% salary support for the first six weeks of the first approved leave and continuation of health and disability benefits [6]. The ABPS additionally permits up to 12 weeks of personal leave for maternity, paternity, family, medical, foster care, adoption, or elective rotations, which may be distributed throughout residency [7]. Our institution provides 10 days each of maternity/paternity and sick leave, with the option to use vacation time for additional paid weeks. We advocate for expanded, paid leave and structured coverage plans-potentially utilizing mid-level providers-to minimize strain on coresidents.

Flexible scheduling

Accommodations for prenatal visits, breastfeeding, and milk storage are essential. Difficulties with lactation among surgical trainees have been repeatedly described, including inadequate time and facilities for milk expression and perceptions of insufficient institutional support. Recent systematic-review evidence similarly identifies lactation as a persistent challenge for surgical residents and attending surgeons and advocates for institutional investment in dedicated facilities, protected time, and supportive policies [8]. Portable breast pumps and secure refrigeration have made it feasible for residents to maintain breastfeeding schedules without compromising clinical duties.

Family-friendly culture

Culture is decisive. Our program leadership openly values family life and encourages residents to start families early if they wish. Over six years, our program has celebrated 22 births-15 within an 18-month span. Residents of both genders have become parents, and children are welcomed at appropriate social events.

Peer support and mentorship

Peer mentorship provides invaluable reassurance. Residents share maternity clothing, childcare advice, and baby items, forming a community of mutual aid. Rangel et al. reported that nearly half of pregnant surgeons operating more than 12 hours weekly experienced complications, underscoring the need for supportive systems that mitigate risks [2].

Inclusion and reintegration

Pregnancy should not interrupt surgical growth. With scheduling flexibility, residents can continue active participation until delivery and reintegrate seamlessly postpartum. Lactation breaks during in-service examinations, for example, are feasible with advance planning.

Childcare and financial support

Childcare remains a significant financial and logistical barrier [9]. Wallace et al. found that 100% of parents in plastic surgery residencies reported childcare as a financial burden, with 63% citing inadequate program flexibility, and only 3% felt that their institution provided services to arrange adequate childcare [3]. Women were also more likely than men to report childcare-related work disruptions. These findings are consistent with broader GME data identifying childcare access, affordability, and sufficient hours of coverage as major challenges for resident parents [4]. Shared childcare models, institutional daycare with extended hours, backup-care programs, and financial assistance could therefore represent meaningful investments in trainee equity and retention.

Recent evidence suggests that institutional policies and culture may influence family-planning decisions among plastic surgery trainees. Gates-Tanzer et al. surveyed 130 plastic surgery trainees and found that more than 70% did not feel they had adequate time for leave; female trainees reported additional barriers related to pregnancy, having a partner in training, and concern about burdening coresidents. Importantly, perceived program support for family planning influenced the decision to apply to plastic surgery residency [5]. These findings reinforce our central argument: family-friendly policies are not merely accommodations for individual residents but may also influence recruitment, specialty choice, and the sustainability of the surgical workforce.

Leadership and role models

Visible role models-especially women in leadership-may help normalize family life within surgery and counter perceptions that parenthood is incompatible with academic and career advancement. Mentors who have navigated pregnancy and parenthood themselves may also be particularly well positioned to advocate for reasonable accommodations and career continuity. Importantly, supporting parental leave need not be viewed as compromising professional productivity: a study examining surgeon productivity before and after leave found no significant reduction in operative volume or relative value units following maternity leave [10].

Programs that genuinely support residents’ family goals strengthen the specialty by attracting and retaining diverse, motivated applicants. As one resident’s child proudly declared, she wants to be “a mom plastic surgeon” when she grows up-a reminder that today’s inclusivity cultivates tomorrow’s leaders. More broadly, women remain underrepresented at senior levels of academic surgery despite increasing representation in medical training, and studies identify institutional culture, mentorship, leadership relationships, and family responsibilities as contributors to persistent gender inequities [11-13]. The reported “leaky pipeline” of women leaving academic plastic surgery after motherhood need not be inevitable. Although parenthood should not be assumed to be the sole cause of this disparity, studies have identified institutional culture, mentorship, leadership relationships, and family responsibilities as contributors to persistent gender inequities [11-13]. True progress therefore requires more than encouraging individual resilience; it requires sustained institutional attention to the structural conditions that influence retention and advancement [14].

There is never a perfect time to start a family, but there can be a perfect environment-one that values both surgeon and parent. As a specialty dedicated to restoration and renewal, plastic surgery is well suited to lead in rewriting the narrative of family in surgical training.
